# Supplementary figures and images for: A single-cell atlas of the Culex tarsalis midgut during West Nile virus infection
Source: PLoS Pathog. 2025 Jan 27;21(1):e1012855. doi: 10.1371/journal.ppat.1012855 (PMC11793825; doi:10.1371/journal.ppat.1012855)

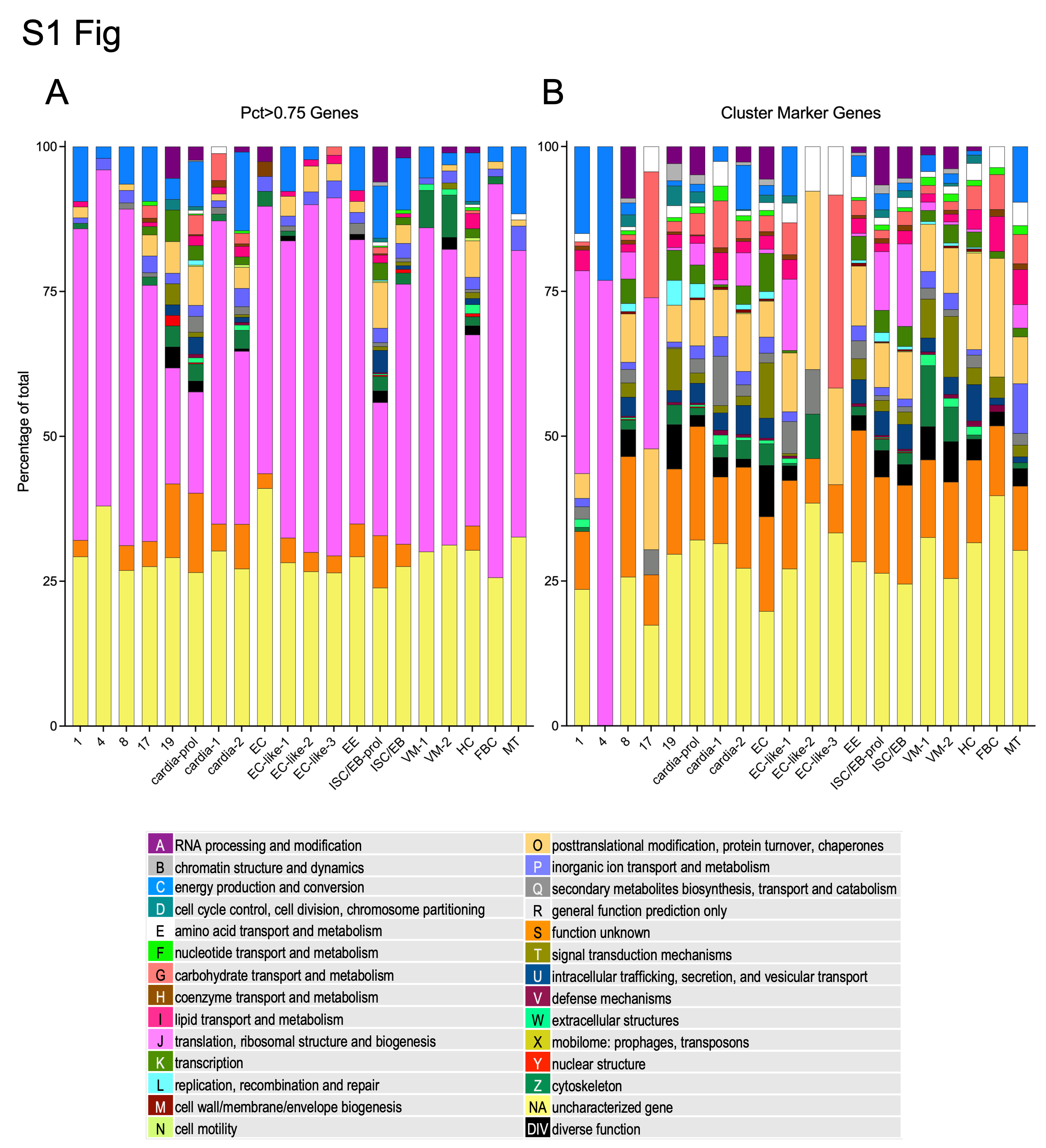

Supplement: S1 Fig — Cluster of orthologous gene (COG) profiles for (A) genes expressed in ≥75% of cells in each cluster and (B) cluster marker genes were visualized as percentage of total for each cluster/population. Colors represent COG notation A-Z, NA and DIV as shown in the notation key embedded in the figure. Where applicable, marker gene COG profiles were derived from cluster markers that are conserved between infection conditions. (TIF) [file ppat.1012855.s001.tiff]

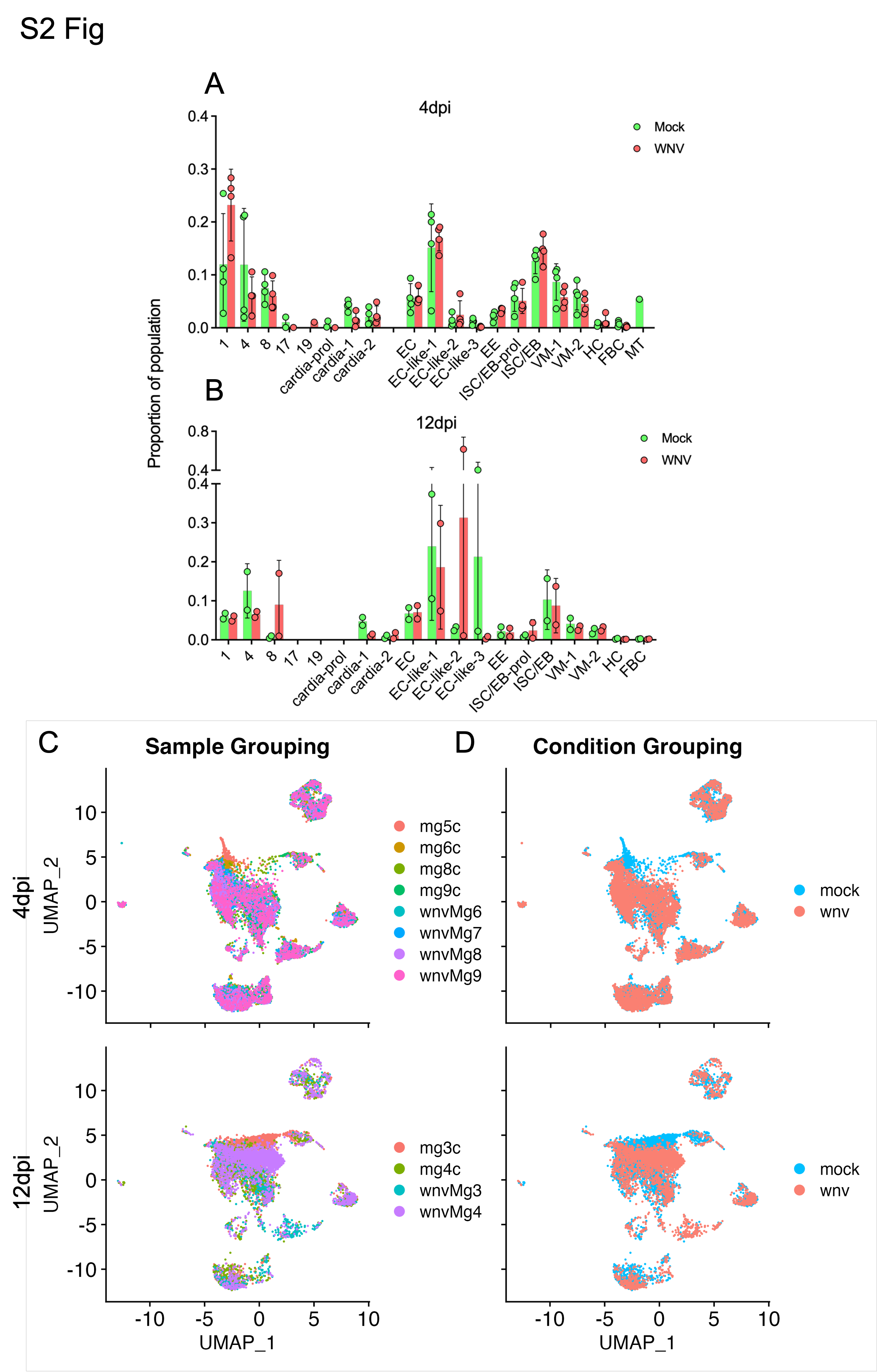

Supplement: S2 Fig — Proportion of the total population comprised by each cluster compared between mock and WNV-infected conditions at 4dpi (A) and 12dpi (B). Only significant comparisons shown. Significance determined by multiple unpaired t-tests. Bar = mean, error bars = SD. (C) Cluster grouping and composition by sample—mock and infected samples both plotted. Different colors denote different samples. (D) Cluster grouping and composition by infection condition. Salmon = mock, blue = WNV-infected. (TIF) [file ppat.1012855.s002.tiff]

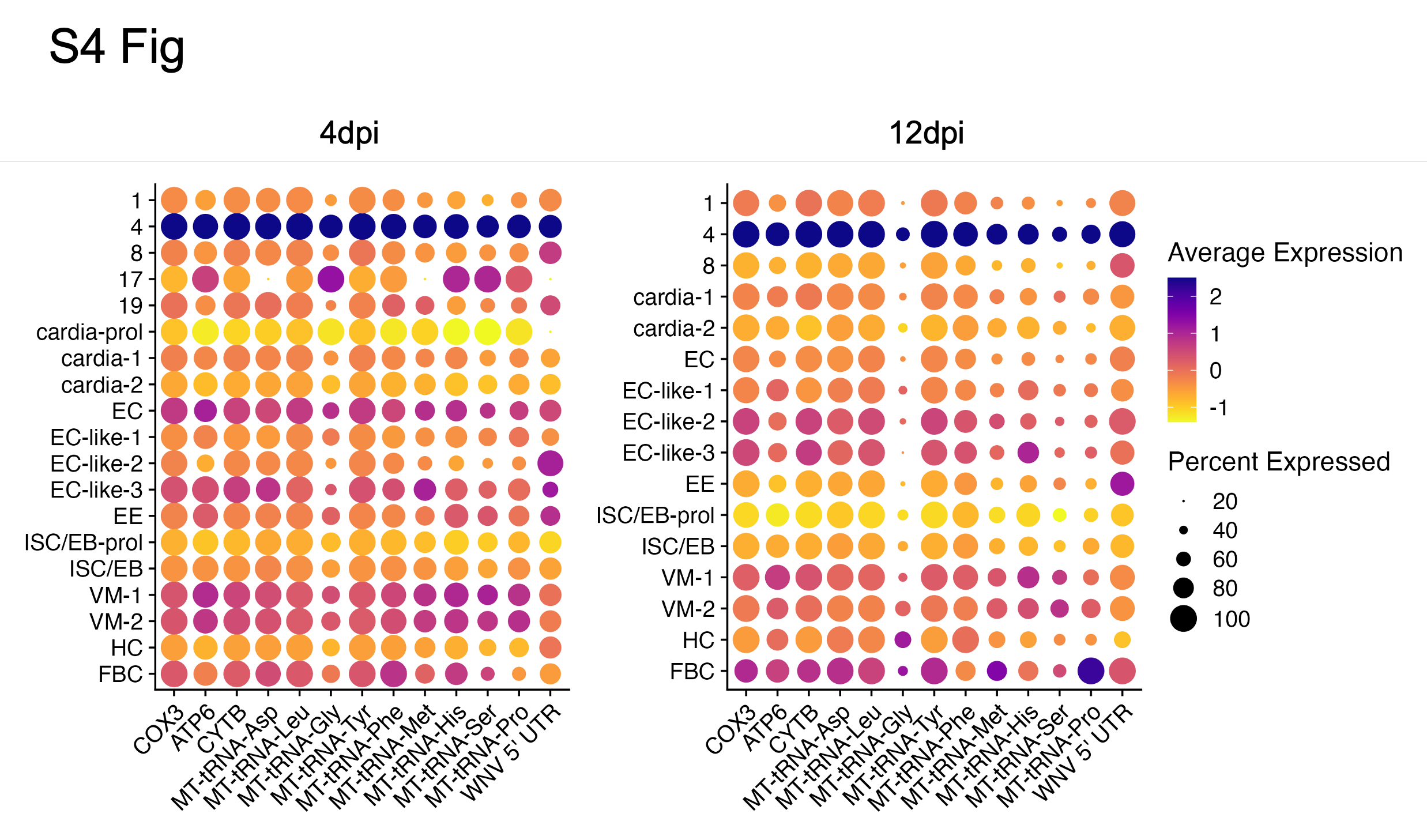

Supplement: S4 Fig — Dot size denotes percent of cells expressing each gene, color denotes scaled gene expression. “MT-“ prefix was added to tRNA gene names in this figure for clarity. Mitochondrial tRNAs were not included in mitochondrial gene estimation for QC filtering. Plots were derived from only WNV-infected samples. (TIF) [file ppat.1012855.s004.tiff]

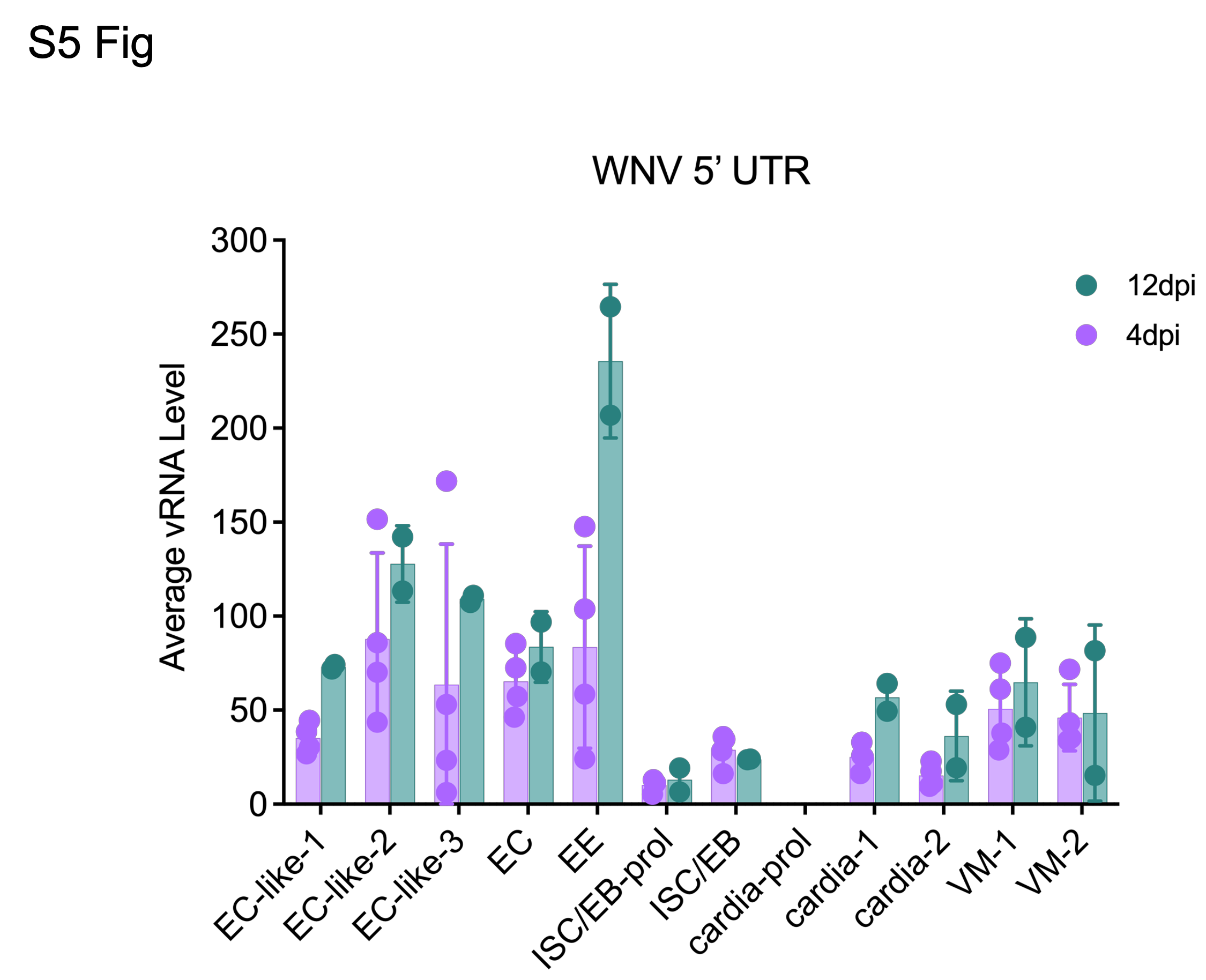

Supplement: S5 Fig — Purple = 4dpi, green = 12dpi. Points within bars denote replicate values, bar denotes mean of replicate values. Error bars = SD. (TIF) [file ppat.1012855.s005.tiff]

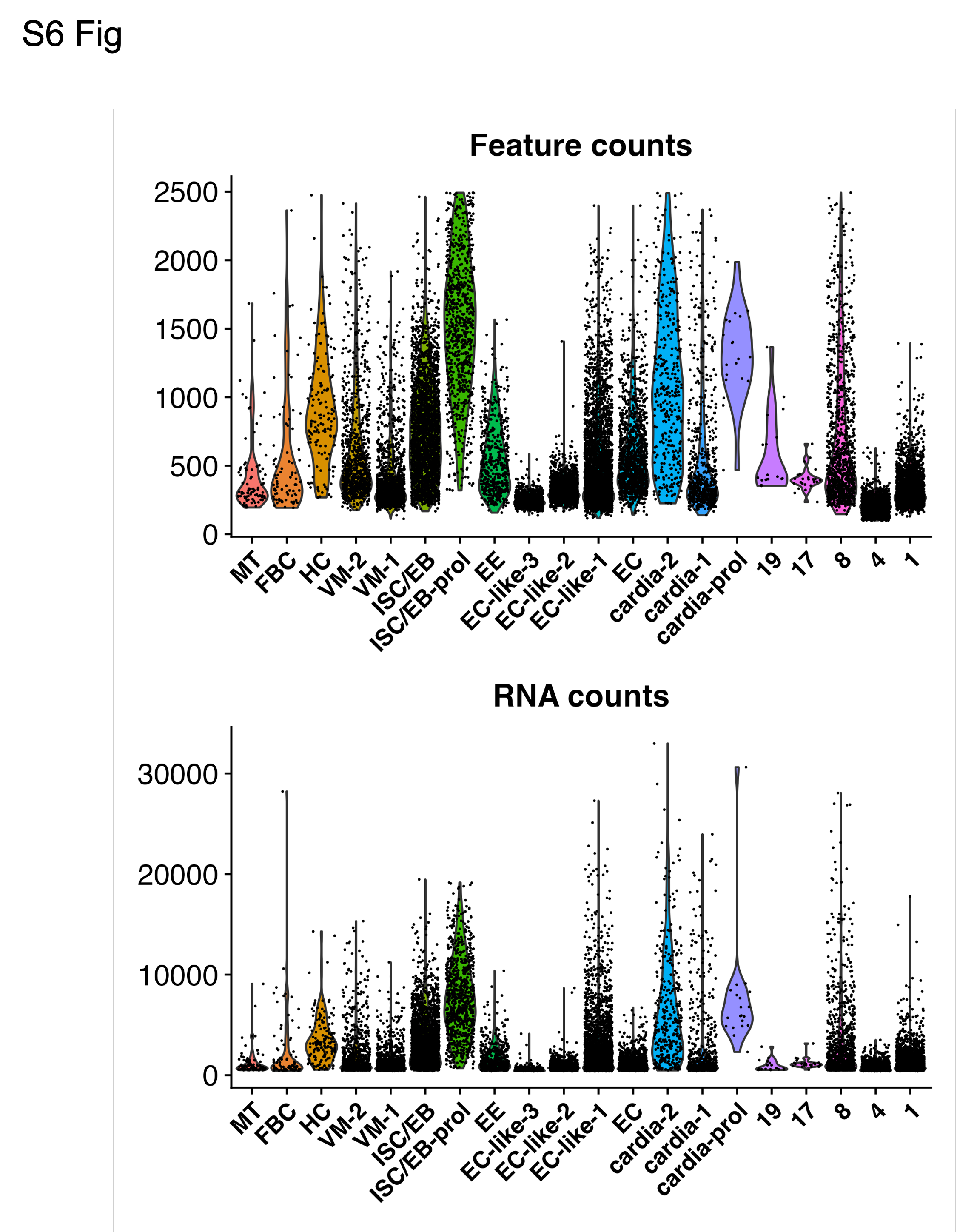

Supplement: S6 Fig — Feature counts and RNA counts for individual cells within clusters. (TIF) [file ppat.1012855.s006.tiff]

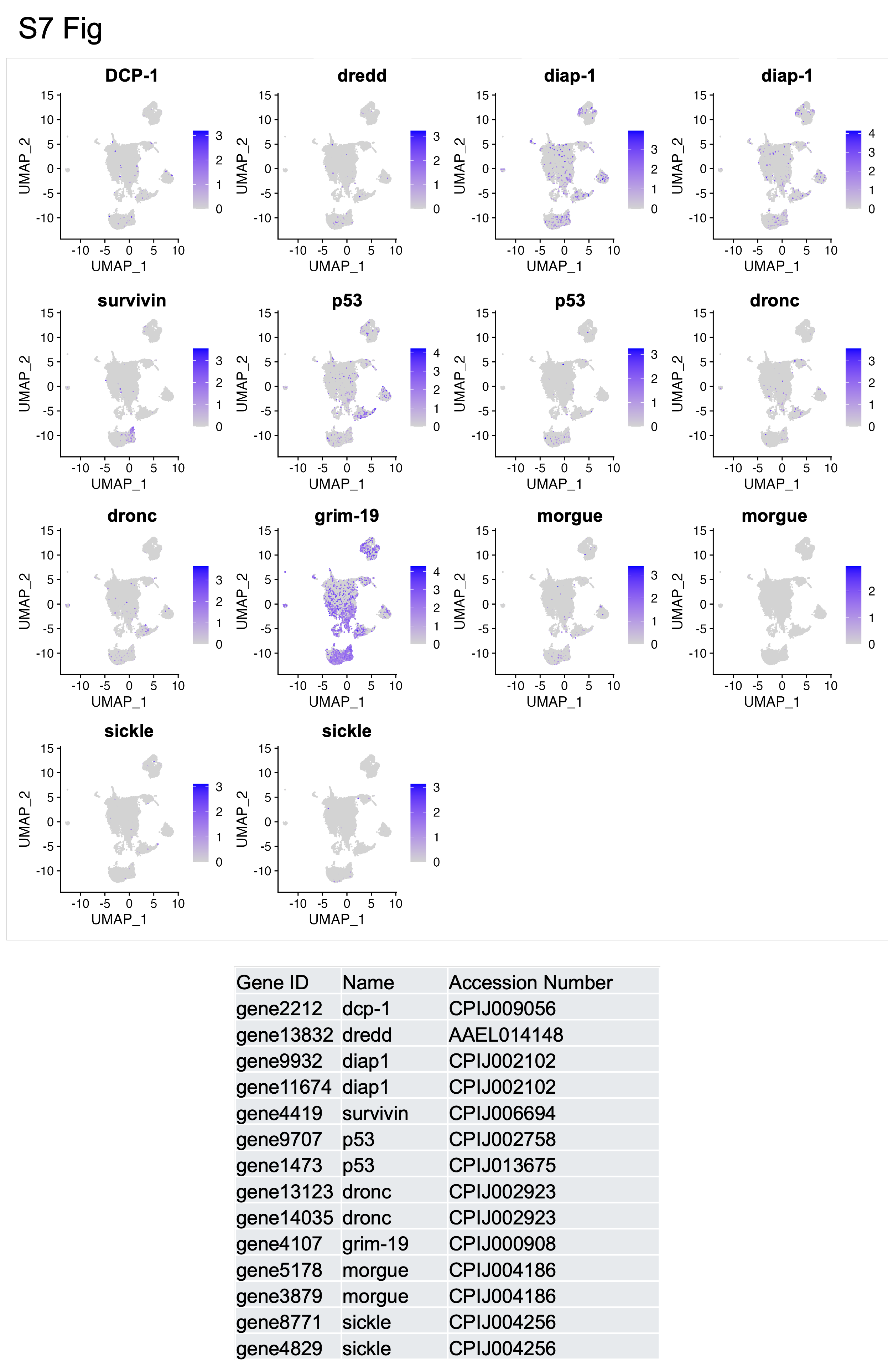

Supplement: S7 Fig — Expression of apoptotic and anti-apoptotic genes visualized in the total population via UMAP feature plot. Color in feature map denotes expression level. (TIF) [file ppat.1012855.s007.tiff]

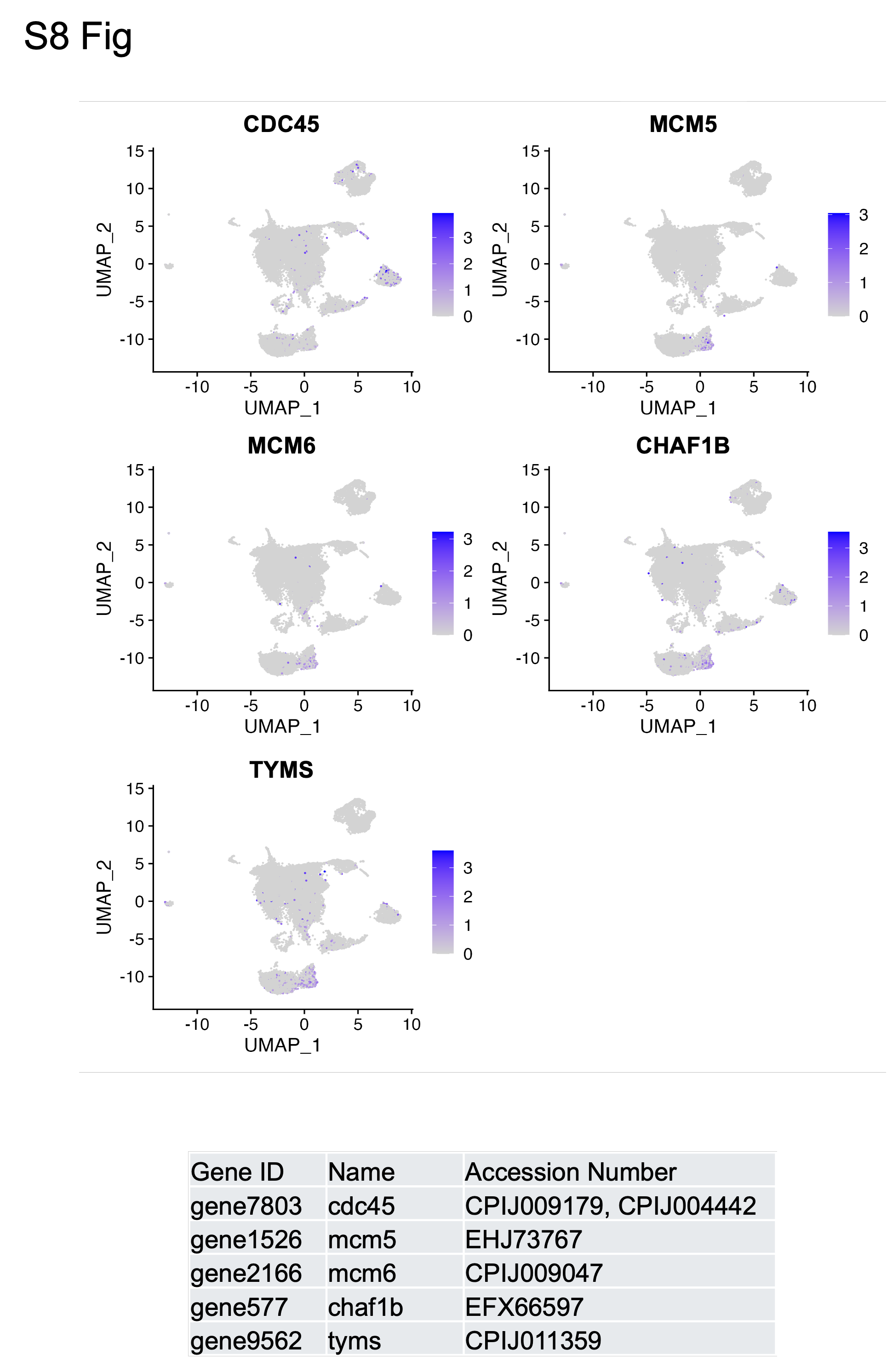

Supplement: S8 Fig — Expression of S phase gene markers visualized in the total population via UMAP feature plot. Color in feature map denotes expression level. (TIF) [file ppat.1012855.s008.tiff]

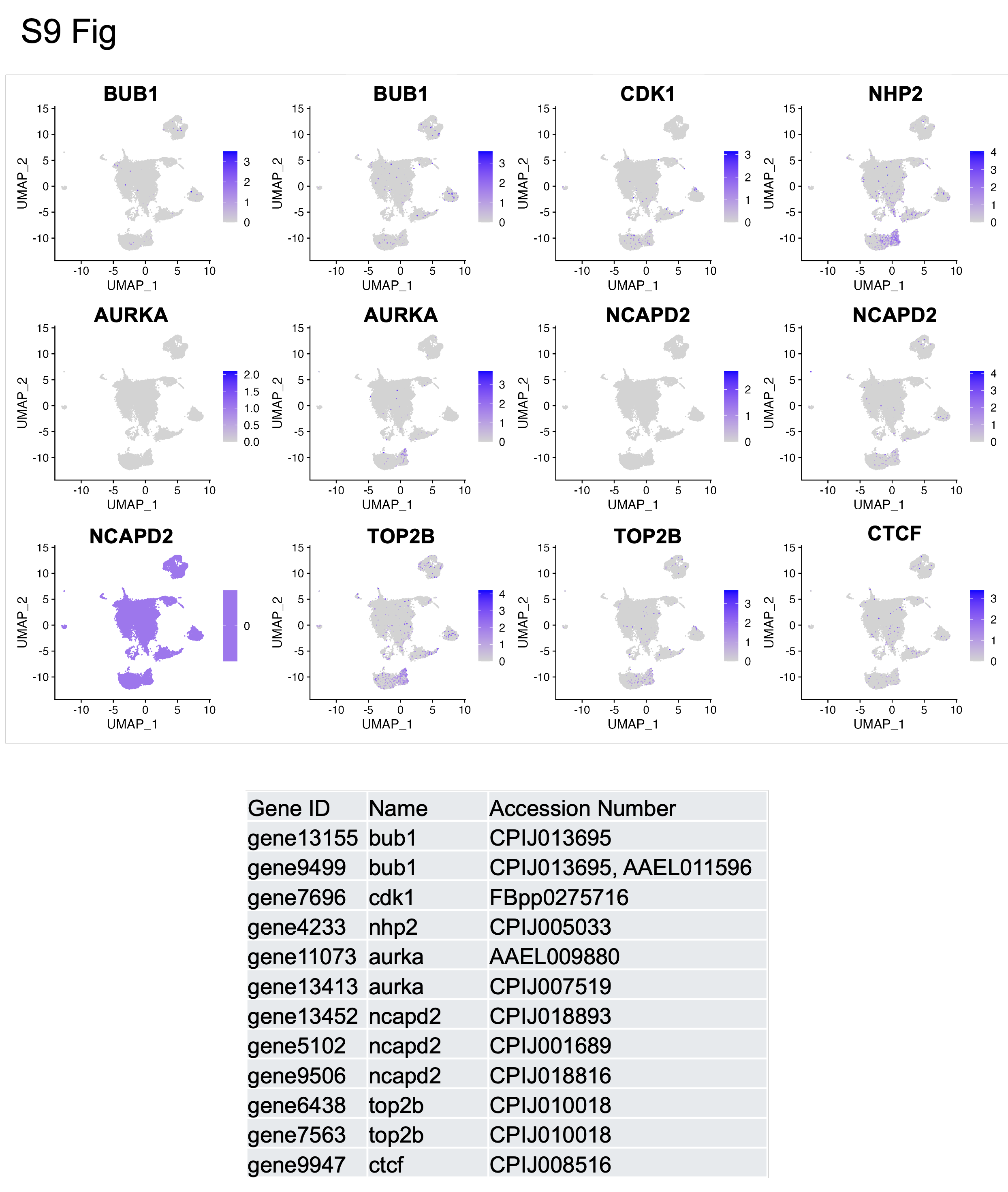

Supplement: S9 Fig — Expression of G2/M phase gene markers visualized in the total population via UMAP feature plot. Color in feature map denotes expression level. (TIF) [file ppat.1012855.s009.tiff]

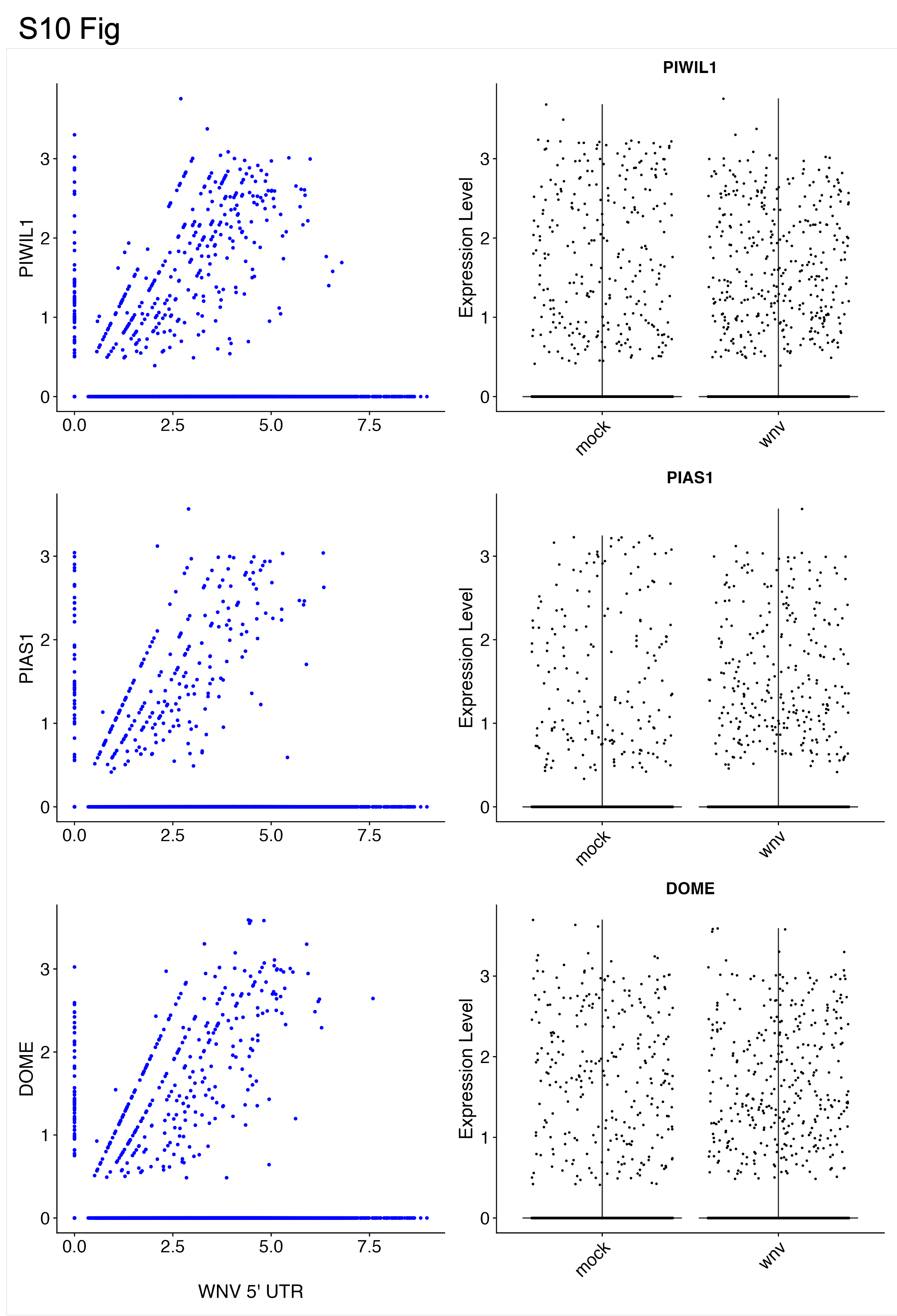

Supplement: S10 Fig — Correlation between vRNA and 3 of the most highly correlated immune genes (as determined by scLink) confirmed by feature scatter. Equivalent expression levels of immune genes between mock and WNV-infected conditions confirmed by violin plot. Scatter plots derived from only WNV-infected replicates. (TIF) [file ppat.1012855.s010.tiff]

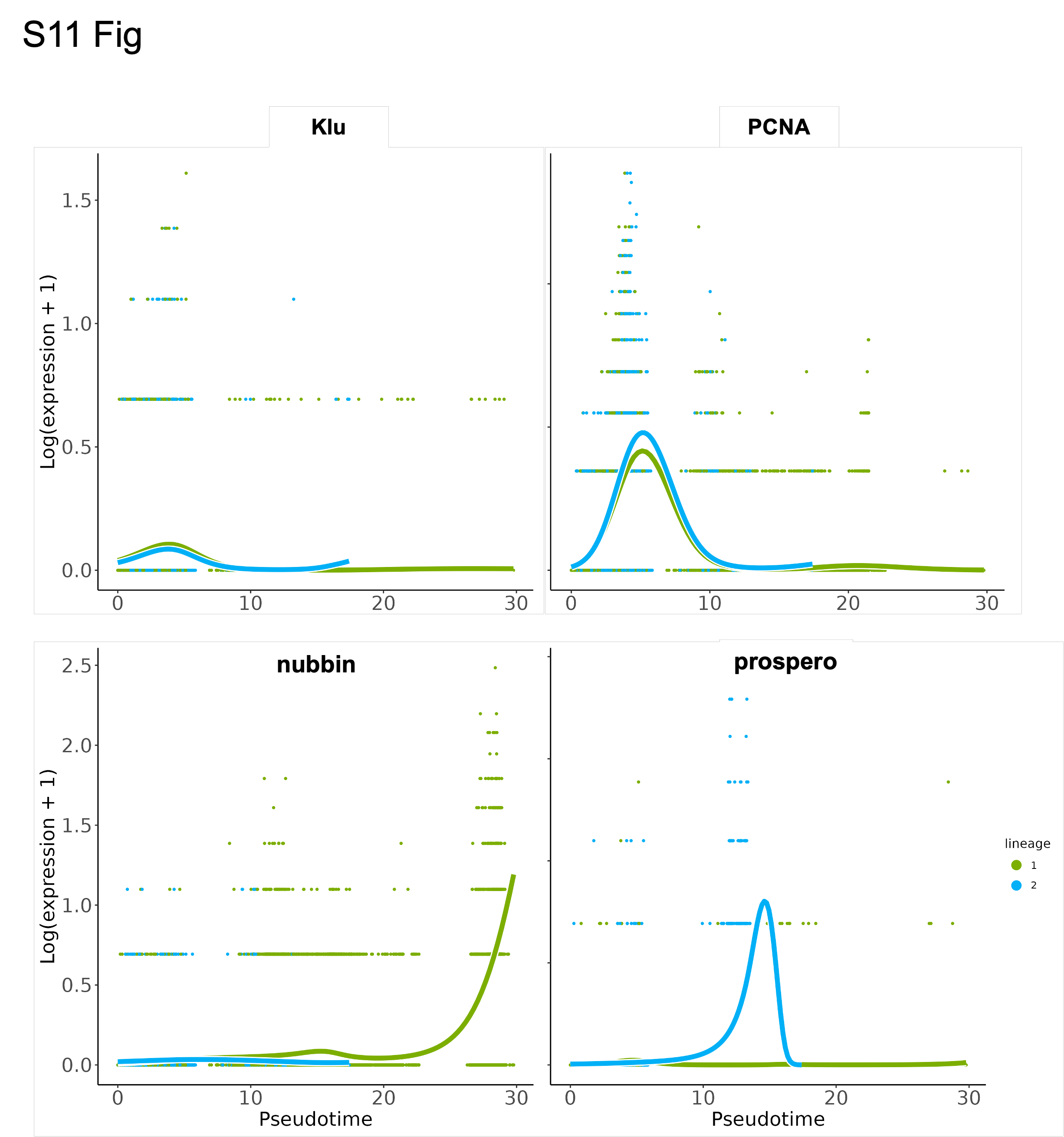

Supplement: S11 Fig — Lineage 1 (green–EC lineage) and 2 (blue–EE lineage) displayed expression of klumpfuss (Klu), the canonical marker for enteroblasts, prior to differentiation. PCNA expression was associated with the progression of each lineage into the proliferating ISC/EB population. The canonical marker for ECs (POU2F1/nubbin) was enriched upon differentiation of lineage 1 into ECs. The canonical marker for EEs (PROX1/prospero) was enriched upon differentiation of lineage 2 into EEs. (TIF) [file ppat.1012855.s011.tiff]

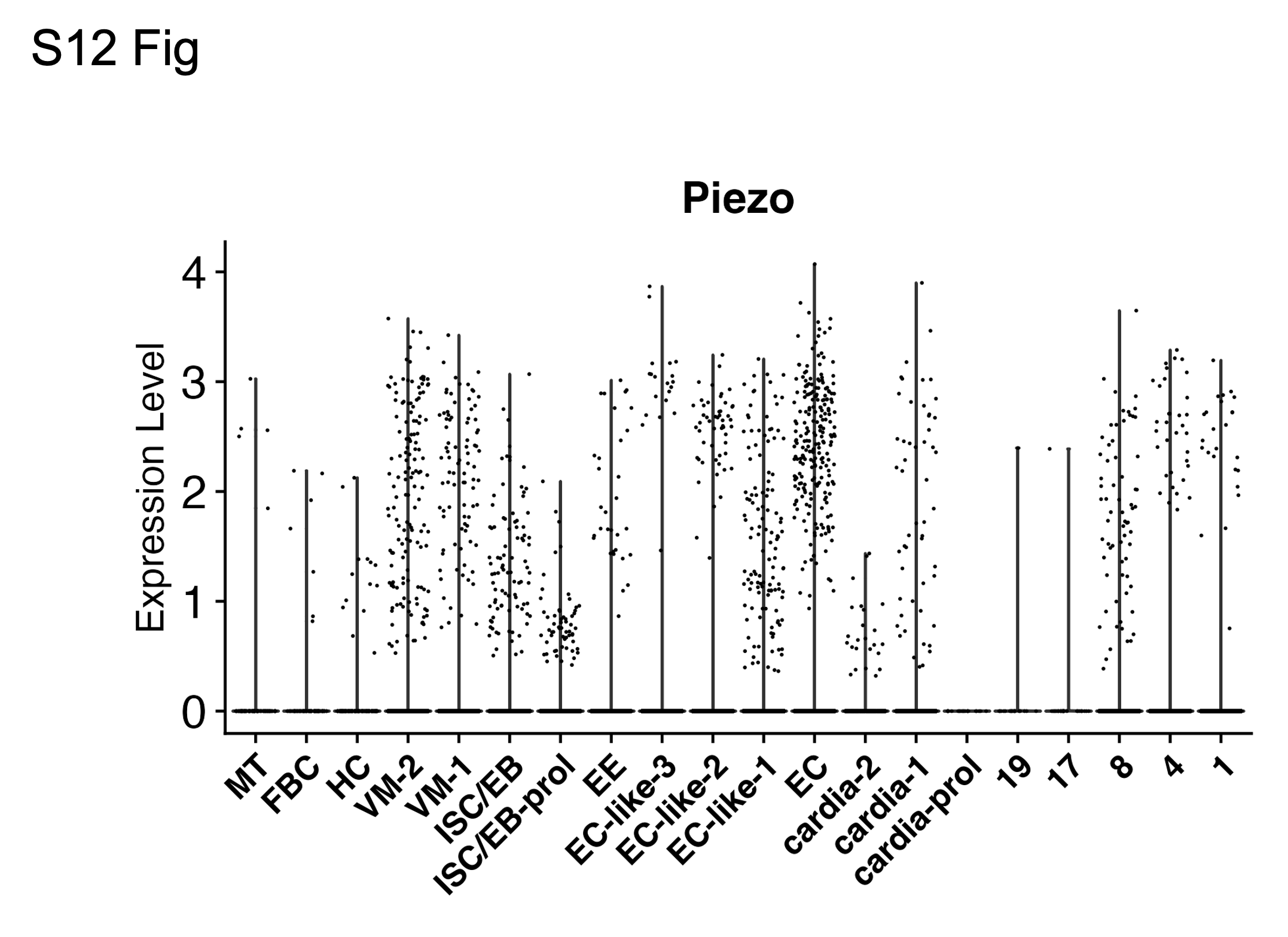

Supplement: S12 Fig — Expression of the EEP gene piezo in individual cells grouped by cluster. (TIF) [file ppat.1012855.s012.tiff]
