# Supplementary material for: A single-cell atlas of the Culex tarsalis midgut during West Nile virus infection
Source: PLoS Pathog. 2025 Jan 27;21(1):e1012855. doi: 10.1371/journal.ppat.1012855 (PMC11793825; doi:10.1371/journal.ppat.1012855)
Supplement: S3 Fig — Visualizing expression of (A) neuroendocrine and secretory genes, (B) proliferation and mitotic marker genes, and (C) HC class marker genes across the total population to determine specificity of expression. Violin plots show expression level in individual cells grouped by cluster. (D) Gene IDs and accession numbers for all genes described in this figure. (PPTX) [file ppat.1012855.s003.pptx]

## Slide 1
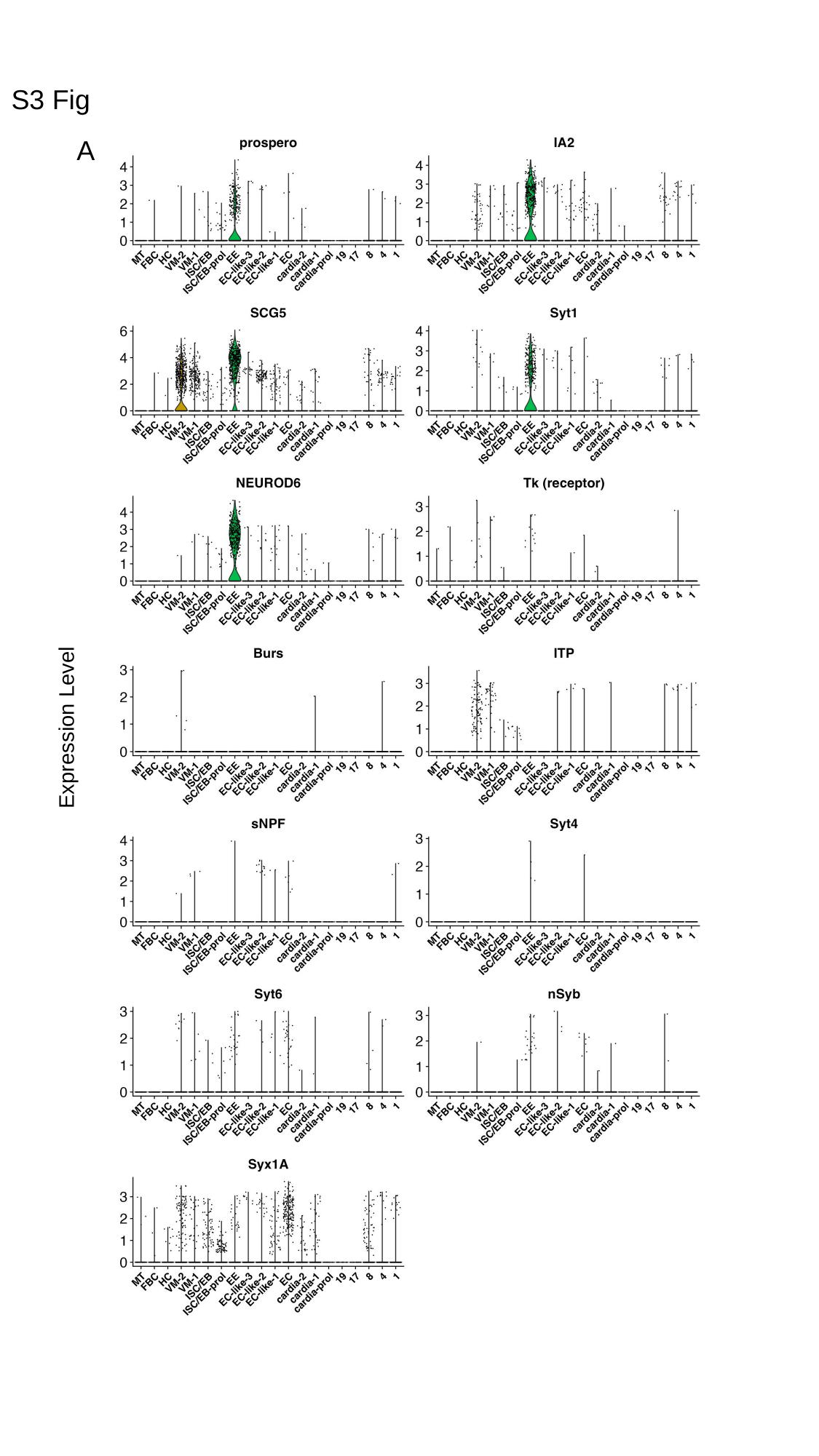

S3 Fig
A
Expression Level

## Slide 2
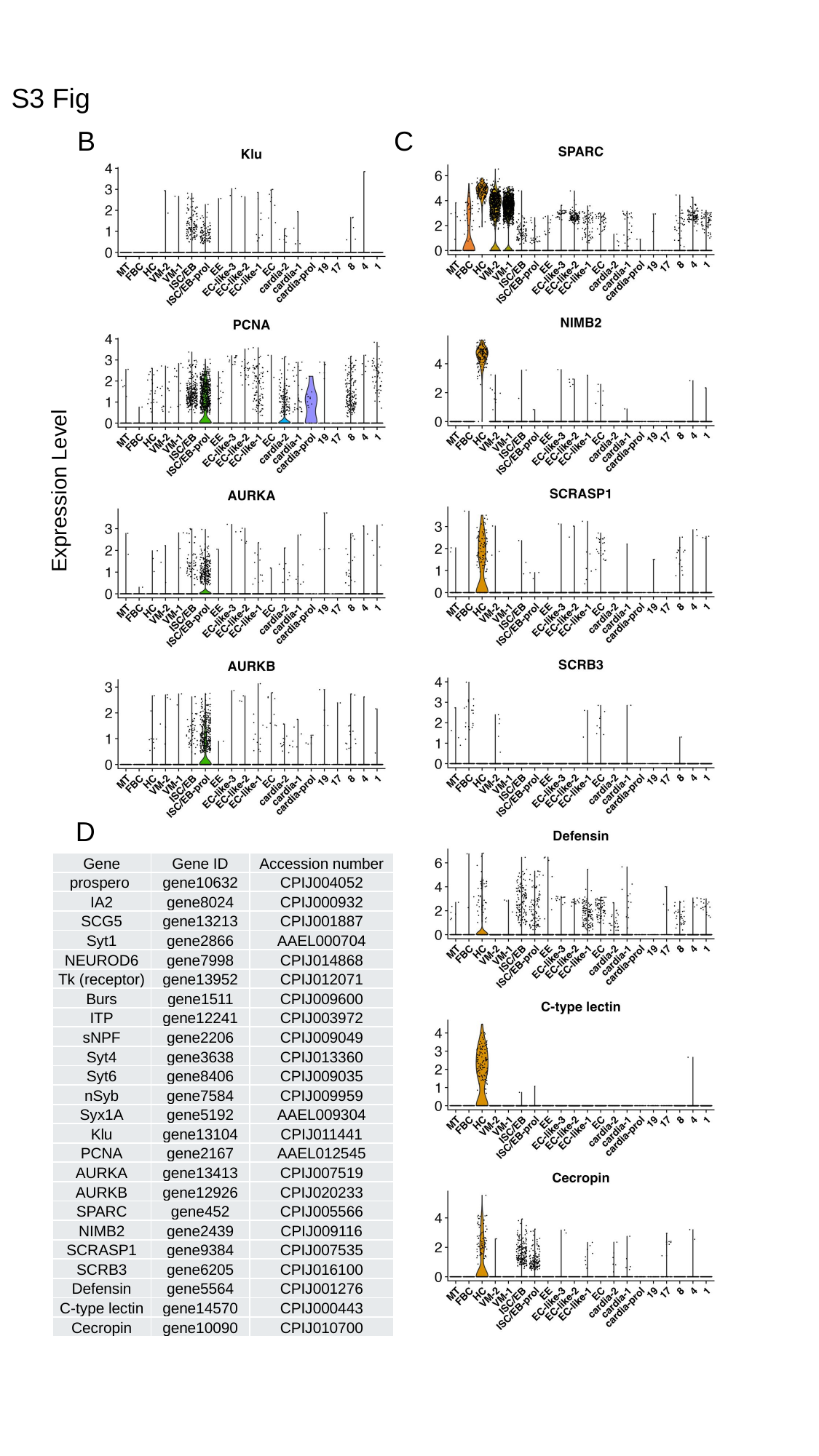

S3 Fig
B
C
Expression Level
D
| Gene | Gene ID | Accession number |
| --- | --- | --- |
| prospero | gene10632 | CPIJ004052 |
| IA2 | gene8024 | CPIJ000932 |
| SCG5 | gene13213 | CPIJ001887 |
| Syt1 | gene2866 | AAEL000704 |
| NEUROD6 | gene7998 | CPIJ014868 |
| Tk (receptor) | gene13952 | CPIJ012071 |
| Burs | gene1511 | CPIJ009600 |
| ITP | gene12241 | CPIJ003972 |
| sNPF | gene2206 | CPIJ009049 |
| Syt4 | gene3638 | CPIJ013360 |
| Syt6 | gene8406 | CPIJ009035 |
| nSyb | gene7584 | CPIJ009959 |
| Syx1A | gene5192 | AAEL009304 |
| Klu | gene13104 | CPIJ011441 |
| PCNA | gene2167 | AAEL012545 |
| AURKA | gene13413 | CPIJ007519 |
| AURKB | gene12926 | CPIJ020233 |
| SPARC | gene452 | CPIJ005566 |
| NIMB2 | gene2439 | CPIJ009116 |
| SCRASP1 | gene9384 | CPIJ007535 |
| SCRB3 | gene6205 | CPIJ016100 |
| Defensin | gene5564 | CPIJ001276 |
| C-type lectin | gene14570 | CPIJ000443 |
| Cecropin | gene10090 | CPIJ010700 |
